# Smartphone use and addiction during the coronavirus disease 2019 (COVID-19) pandemic: cohort study on 184 Italian children and adolescents

**DOI:** 10.1186/s13052-021-01102-8

**Published:** 2021-07-02

**Authors:** Gregorio Serra, Lucia Lo Scalzo, Mario Giuffrè, Pietro Ferrara, Giovanni Corsello

**Affiliations:** 1 Department of Health Promotion, Mother and Child Care, Internal Medicine and Medical Specialties “G. D’Alessandro”, University of Palermo, Palermo, Italy; 2 Unit of Pediatrics, Campus Bio-Medico University, Rome, Italy

**Keywords:** Smartphone, Addiction, COVID-19, School-age children, Italy

## Abstract

**Background:**

The lives of many children and adolescents are today increasingly influenced by new technological devices, including smartphones. The coronavirus disease 2019 (COVID-19) pandemic occurred in a time of outstanding scientific progress and global digitalization. Young people had relevant adverse psychological and behavioral effects due to the COVID-19 pandemic, mainly related to infection control measures, which led them to spend more time at home and with major use of technological tools. The goal this study proposes is to evaluate health and social outcomes of smartphone overuse among Italian children and adolescents during the COVID-19 pandemic, analyzing patterns and aims of utilization, as well as the eventual presence and degree of addiction.

**Methods:**

This study was based on a self-report and anonymous questionnaire, which was administered to 184 Italian school-age (6–18 years) children and adolescents during the second wave of the COVID-19 pandemic. The test was electronically (email, whatsapp) explained and sent by pediatricians either directly to older children (middle and high school), or indirectly, through the help of teachers, to younger ones (primary school). All participants spontaneously and voluntarily joined the present study. The survey was made by 4 sections, and designed to know and outline modalities (frequency, patterns and aims) of smartphone use, adverse outcomes, and related parental behaviors, also in order to reveal the eventual occurrence and degree of addiction. The same information, related to the pre-epidemic period, was also investigated and analyzed.

**Results:**

The data obtained revealed a significantly greater adhesion to the questionnaire by females, likely reflecting higher attention and interest than boys to initiatives relating to health education. Our study showed more frequent smartphone use among Italian children and adolescents during the COVID-19 pandemic, compared to the pre-epidemic period. This may be related to the social distancing measures adopted during the months under investigation. The present survey also outlined the changing patterns and aims in the use of smartphones among young people, which allowed to limit some effects of the crisis. Indeed, they were used for human connection, learning and entertainment, providing psychological and social support. Finally, it was observed a significant increase of overuse and addiction. This led to many clinical (sleep, ocular and musculoskeletal disorders), psychological (distraction, mood modification, loss of interest) and social (superficial approach to learning, isolation) unfavorable outcomes.

**Conclusions:**

Pediatricians and health care professionals should be aware of the potential risks related to inappropriate use of smartphones. They should monitor, in cooperation with parents, possible associated adverse effects, in order to early recognize signs and symptoms suggestive, or at high risk, for addiction. They must carry out, as well, the necessary interventions to prevent and/or lower the detrimental impact of smartphone overuse on children and adolescents’ health, oriented to sustain adequate physical and psychological development as well as social relationships.

## Background

The lives of many children and adolescents are today increasingly influenced by new technological devices and means of communication (smartphones, tablets, social network) [1]. Specifically, in recent years, smartphone use has rapidly increased worldwide. In Italy, the number of users is constantly growing, and it is estimated to reach in 2025 around 42 million subjects [2]. Its overuse may lead to clinical, psychological and social adverse outcomes [3–5]. Although smartphone addiction is not included in the latest version of the Diagnostic and Statistical Manual for Mental Disorders, research studies and social interventions were conducted to address its related unfavorable effects [6]. The coronavirus disease 2019 (COVID-19) pandemic occurred at a time of global digitalization, characterized by quick connection of people and information anywhere in the world. Children and adolescents had lower incidence and mortality rates of COVID-19 than adults [7, 8]. However, they had relevant adverse psychological and behavioral effects, especially among school-age ones, due to home confinement and other infection control measures, such as social distancing, and closure of schools and shared public spaces [9]. This led them, as well as adults, to spend more time at home and with major use of technological devices [10]. The goal this study proposes is to evaluate health outcomes and social implications of smartphone overuse among Italian children and adolescents during the COVID-19 pandemic, analyzing patterns and aims of utilization, as well as the eventual presence and degree of addiction, and comparing them with those of the pre-epidemic period.

## Methods

### Study setting

COVID-19, the acute respiratory syndrome related to the new coronavirus (SARS-CoV-2) infection, dramatically spread worldwide from China since the end of 2019. It reached Italy, first among European countries, on February 2020. The following further rapid diffusion to other continents on March 11th 2020, led the World Health Organization (WHO) to declare a pandemic. The study took place over a 2-month period, from December 2020 to January 2021. In that time Italy, within the second wave of the pandemic, was divided into three zones - red, orange, yellow- corresponding to three different scenarios of epidemiologic risk [11]. The division of the country into three areas was based on 21 criteria, established by the Italian National Institute of Health. The main indicators included local/regional infection rates and hospital (ordinary and intensive care) occupancy, as well as other factors related to care capacity/outbreak management of the different regional health systems (i.e. availability of health care professionals, effectiveness of the contact tracing system) [12]. Specific strategies to contrast the spread of the disease were taken and tuned, based on the different risk scenarios. Where the overall risk of COVID-19 spread was increased, the movement of people and the economic and social activities were more limited according to the Decrees of the President of the Council of Ministers (DPCM), which varied on a period basis. Teaching and educational activities and services were intermittently carried out in presence for children of infant schools, primary schools and partially of lower secondary schools. For the remaining lower secondary school children and for high school adolescents, distance teaching (online) was the only modality of education which was guaranteed [13].

### Selection of study participants

This study was based on a self-report and anonymous questionnaire, which was administered to Italian school-age (6–18 years) children and adolescents during the second wave of the COVID-19 pandemic. The test was conceived and made by pediatricians of the Mother and Child Department of the University of Palermo (Sicily, Italy). Questions were created in Google Forms. They were electronically (email, whatsapp) explained in detail, together with the aims of this study, and directly sent to older children (middle and high school), or indirectly, through the help of teachers, to younger ones (primary school). The forms were addressed in Sicily and in regions of northern Italy (Piedmont, Lombardy). Among around three hundred questionnaires homogeneously distributed between males and females of different age groups, a total of 185 students replied and participated in the study. All participants spontaneously and voluntarily joined the present survey. Lack of partial and/or total completion of the questionnaire was the only exclusion criterion. Their answers were recorded on a web-based Google sheet form, and then analyzed.

### Data collection

The questionnaire was designed to understand modalities (frequency, patterns and aims) of smartphone use, its impact (adverse effects) on everyday life, and related parental behaviors among children and adolescents, also in order to reveal the eventual occurrence and degree of addiction, before and during the COVID-19 pandemic. It was made by 4 sections.

The first section was about demographic and socioeconomic characteristics, and included the following variables: age, gender, ethnicity, school year, region of residence, family status, parents’ educational level, occupation and income, age at onset of smartphone use.

The second section concerned COVID-19 related questions: family members’ jobs linked to the epidemic (specific occupation), family members and/or friends infected by SARS-CoV-2 or dead due to COVID-19, degree of concern about the pandemic, implementation of preventive and infection control measures, eventual distance (online) learning and impact of the pandemic on education.

The third section included questions on frequency of smartphone use (i.e. time of smartphone use per day, time between wake up and start of use, frequency of night utilization), and about patterns and effects of their utilization before and during the pandemic, evaluated with the following items: “most frequently used functions”, “aim of use”, “adverse effects” and “parental attitude towards smartphone use”. The most frequently used functions included: “telephone call”, “social network (Instagram, Facebook)”, “game”, “education”, “online chat (Whatsapp)”, “photos”, “videos” and “music”. To analyze the aims of smartphone use, we included the following items: “boredom”, “habit”, “pleasure”, “game”, “communication”, “learning”, “stress relief” and “adaptation to others”. The answers on adverse effects included: “superficial approach to learning”, “distraction”, “mood modification”, “loss of interest”, “isolation”, “sleep disturbances”, “ocular alterations” and “musculoskeletal disorders”. Questions on parental attitude regarding smartphone use included the following items: “surveillance”, “restriction”, “punishment”, “permission” and “explanation”. Participants gave to each of these four items a score, ranging from 1 (never) to 5 (always).

The fourth section was related to the evaluation of eventual occurrence and degree of smartphone addiction, measured by the Italian Smartphone Addiction Scale Short Version (SAS-SV) [14]. It is a well-validated specific questionnaire, which contains 10 questions including daily-life disturbance, positive anticipation, withdrawal, cyberspace-oriented relationship, overuse and tolerance (Table 1).

**Table 1:** Italian Smartphone Addiction Scale Short Version (modified from De Pasquale et al., 2017) [14]

1	Missing planned work due to smartphone use
2	Having a hard time concentrating in class, while doing assignments, or while working due to smartphone use
3	Feeling pain in the wrists or at the back of the neck while using a smartphone
4	Will not be able to stand not having a smartphone
5	Feeling impatient and fretful when I am not holding my smartphone
6	Having my smartphone in my mind even when I am not using it
7	I will never give up using my smartphone even when my daily life is already greatly affected by it
8	Constantly checking my smartphone so as not to miss conversations between other people on WhatsApp, Facebook or Instagram
9	Using my smartphone longer than I had intended
10	The people around me tell me that I use my smartphone too much

Participants expressed their opinion for each item over a 6-point scale, ranging from 1 (strongly disagree) to 6 (strongly agree). A different normal range is identified for males and females. Males are considered addicted if scores are higher than 31. High risk of addiction is present with scores between 22 and 31. Females are addicted if scores are higher than 33, and at high risk with scores between 22 and 33 [15].

### Statistical analysis

We used R version 4.0.4 (R Foundation for Statistical Computing, Vienna, Austria) for data analysis. Simple descriptive statistics were expressed as frequency and percentage for categorical variables, mean and standard deviation (SD) for continuous variables. Paired-samples t-test was used to compare data on patterns of smartphone use in the study population, before and during the COVID-19 pandemic. A *p* value lower than 0.05 was considered statistically significant.

## Results

### Sociodemographic characteristics

Of the 185 study participants, one was excluded (0.5%) because he did not complete the questionnaire. Thus, 184 school-age children and adolescents were included. Among them, 52 (28.3%) were male and 132 (71.7%) female. Their mean age was 14.84 ± 2.73 years. The sample comprised 39 (21.2%) children aged 6–12 years, and 145 (78.8%) adolescents aged 13–18 years. Ten (5.4%) attended primary school, 48 (26.1%) lower secondary school (middle school) and 126 (68.5%) upper secondary school (high school). All study participants were Italian. They came from 3 different regions: 167 (90.8%) Sicily, 15 (8.1%) Piedmont and 2 (1.1%) Lombardy. 152 (82.6%) belonged to urban areas, and 32 (17.4%) to rural ones. One hundred seventy-two (93.5%) lived with married parents, 12 (6.5%) with only one parent, due to parental separation/divorce (7) or death (5). Seventy-one (38.6%) mothers were graduated, 86 (46.7%) had high school education, 26 (14.2%) lower secondary school, and one (0.5%) primary school. 59 (32.1%) fathers were graduated, 88 (47.8%) had high school education, 36 (19.6%) lower secondary school, and one (0.5%) primary school. One hundred participants (54.3%) belonged to double-income families (employed and independent workers), 84 (45.7%) to single-income ones (unemployed/housewives). All respondents owned a smartphone. The average age at owning smartphone was 10.57 ± 1.8 years (Table 2).

**Table 2:** Sociodemographic characteristics of the study population

Variables	N (%)
**Sex**	
Male	52 (28.3%)
Female	132 (71.7%)
**Age (years)**	
Range	6–18
6–12	39 (21.2%)
13–18	145 (78.8%)
Mean ± SD	14.84 ± 2.73
**Educational level**	
Primary school	10 (5.4%)
Middle school	48 (26.1%)
High school	126 (68.5%)
**Region of residence**	
Sicily	167 (90.8%)
Piedmont	15 (8.1%)
Lombardy	2 (1.1%)
**Area of origin**	
Urban	152 (82.6%)
Rural	32 (17.4%)
**Family status**	
Married parents	172 (93.5%)
Separated/divorced parents	7 (3.8%)
Widow parent	5 (2.7%)
**Parents’ educational level**	
**Mother**	
Primary school	1 (0.5%)
Middle school	26 (14.2%)
High school	86 (46.7%)
Graduation	71 (38.6%)
**Father**	
Primary school	1 (0.5%)
Middle school	36 (19.6%)
High school	88 (47.8%)
Graduation	59 (32.1%)
**Family income**	
Double-income	100 (54.3%)
Single-income	84 (45.7%)
**Age at owning smartphone (years)**	
Range	6–15
Mean ± SD	10.57 ± 1.8

*SD* standard deviation

### COVID-19 related data

Twenty-four (13%) participants reported a work related to the epidemic emergency for their family members, 18 of whom referring health care professions (e.g., doctors and nurses).Seventy-six (41.3%) subjects stated that family members and/or friends were infected with SARS-CoV-2. Four (2.2%) adolescents lost a relative due to COVID-19. A total of 83 (45.1%) respondents reported to be very concerned about the pandemic, and 98 (53.3%) averagely worried. One hundred sixty-eight (87%) participants had strictly implemented preventive and control measures (e.g., wash hands, wear mask, avoid public and crowded places) against the epidemic spread. Furthermore, 132 (71.7%) children and adolescents reported that the COVID-19 pandemic affected their learning. A total of 176 (95.7%) students followed distance learning (Table 3).

**Table 3:** COVID-19 related information

Variables	N (%)
**Family members with jobs related to the epidemic**	
Both parents	6 (3.2%)
Father	11 (6%)
Mother	7 (3.8%)
None	160 (87%)
**Family members’ work related to the epidemic emergency**	
Doctor	7 (29.2%)
Nurse	3 (12.5%)
Other health care professional	8 (33.3%)
Different from health care professional	6 (25%)
**Family members and/or friends affected by COVID-19**	
Yes	76 (41.3%)
No	108 (58.7%)
**Relative died for COVID-19**	
Yes	4 (2.2%)
No	180 (97.8%)
**Degree of concern about the pandemic**	
Very concerned	83 (45.1%)
Average	98 (53.3%)
Not concerned	3 (1.6%)
**Implementation of infection prevention and control measures**	
Strictly enforced	168 (91.3%)
Sometimes	13 (7.1%)
Never	3 (1.6%)
**Learning problems due to the pandemic**	
Yes	132 (71.7%)
No	52 (28.3%)
**Distance learning**	
Yes	176 (95.7%)
No	8 (4.3%)

### Smartphone use before and during the COVID-19 pandemic

Average time per day of smartphone use during the pandemic was higher than before: 122 (66.3%) participants spent more than 4 h per day during the COVID-19 pandemic, while those before the pandemic were 30 (16.3%). Time between wake up and start of use was lower during the pandemic, with 104 (56.5%) subjects reporting their first use within 5 min after wake up, compared to 47 (25.5%) during the pre-epidemic period. Frequency of night utilization during the pandemic was also increased than before: 103 (56%) children and adolescents used smartphone after midnight at least 3 times per week, while those during the pre-epidemic period were 56 (30.4%) (Table 4).

**Table 4:** Frequency of smartphone use before and during the COVID-19 pandemic

Frequency of smartphone use	Before the pandemic N (%)	During the pandemic N (%)
**Time per day**		
< 1 h	13 (7.1%)	3 (1.6%)
≥ 1 h < 2 h	76 (41.3%)	10 (5.4%)
≥ 2 h < 4 h	65 (35.3%)	49 (26.6%)
≥ 4 h	30 (16.3%)	122 (66.3%)
**Time between wake up and start of use**		
5 min	47 (25.5%)	104 (56.5%)
> 5 min ≤ 30 min	87 (47.3%)	53 (28.8%)
> 30 min ≤ 60 min	26 (14.1%)	11 (6%)
> 60 min	24 (13.1%)	16 (8.7%)
**Frequency of night utilization**		
Never	53 (28.8%)	31 (16.8%)
Once per week	32 (17.4%)	15 (8.2%)
Twice per week	43 (23.4%)	35 (19%)
≥ 3 times per week	56 (30.4%)	103 (56%)

#### Most frequently used functions

During the COVID-19 pandemic, the participants used more frequently than before the following functions: “telephone call”, “social network (Instagram, Facebook)”, “game”, “education”, “online chat (Whatsapp)”, “music” and “videos”. Conversely, for “photos” a statistically significant difference was not observed (Table 5).

**Table 5:** Patterns of smartphone use and related adverse effects and parental behavior, before and during the COVID-19 pandemic

Items	Range	Before the pandemic Mean ± SD	During the pandemic Mean ± SD	P value
**Most frequently used functions**				
Telephone calls	1–5	3.33 ± 1.09	3.80 ± 1.16	< 0.001^a^
Social network (Instagram, Facebook, etc.)	1–5	3.81 ± 1.27	4.07 ± 1.20	< 0.001^a^
Game	1–5	2.57 ± 1.20	2.85 ± 1.43	0.001^a^
Education	1–5	2.87 ± 1.26	4.13 ± 1.05	< 0.001^a^
Online chat (Whatsapp, etc.)	1–5	3.32 ± 1.53	3.61 ± 1.63	< 0.001^a^
Photos	1–5	3.29 ± 1.21	3.15 ± 1.24	0.1
Videos	1–5	3.43 ± 1.20	3.70 ± 1.31	0.002^a^
Music	1–5	3.79 ± 1.22	3.94 ± 1.25	0.043^a^
**Aim of use**				
Boredom	1–5	3.32 ± 1.10	3.99 ± 1.29	< 0.001^a^
Habit	1–5	3.32 ± 1.12	3.65 ± 1.24	< 0.001^a^
Pleasure	1–5	3.47 ± 1.10	3.59 ± 1.20	0.17
Game	1–5	2.55 ± 1.28	2.82 ± 1.48	0.001^a^
Communication	1–5	4.21 ± 0.93	4.53 ± 0.80	< 0.001^a^
Learning	1–5	2.99 ± 1.09	4.16 ± 1.03	< 0.001^a^
Stress relief	1–5	2.95 ± 1.33	3.34 ± 1.39	< 0.001^a^
Adaptation to others	1–5	2.37 ± 1.23	2.52 ± 1.40	0.082
**Adverse effects**				
Superficial approach to learning	1–5	2.01 ± 1.04	2.63 ± 1.34	< 0.001^a^
Distraction	1–5	2.80 ± 1.06	3.32 ± 1.33	< 0.001^a^
Mood modification	1–5	2.23 ± 1.13	2.85 ± 1.44	< 0.001^a^
Loss of interest	1–5	2.21 ± 1.10	2.80 ± 1.39	< 0.001^a^
Isolation	1–5	1.94 ± 1.09	2.57 ± 1.48	< 0.001^a^
Sleep disturbances	1–5	2.29 ± 1.29	2.88 ± 1.35	< 0.001^a^
Ocular alterations	1–5	1.94 ± 1.07	2.78 ± 1.51	< 0.001^a^
Musculoskeletal disorders	1–5	1.83 ± 1.07	2.74 ± 1.54	< 0.001^a^
**Parental attitude**				
Surveillance	1–5	2.61 ± 1.29	2.52 ± 1.36	0.178
Restriction	1–5	2.31 ± 1.26	2.32 ± 1.33	0.898
Punishment	1–5	2.08 ± 1.18	1.98 ± 1.29	0.0972
Permission	1–5	3.17 ± 1.14	3.43 ± 1.23	< 0.001^a^
Explanation	1–5	3.59 ± 1.22	3.70 ± 1.26	0.1

*SD* standard deviation

^a^ Statistically significant difference comparing the COVID-19 pandemic with the pre-epidemic period

#### Aim of use

The respondents did not show a difference in the aims of use “pleasure” and “adaptation to others”, before and during the COVID-19 pandemic. Conversely, they indicated as aims of use more frequently reported the following ones: “boredom”, “habit”, “game”, “communication”, “learning” and “stress relief” (Table 5).

#### Adverse effects

All of the following adverse effects (clinical, psychological/behavioral, social) related to smartphone use were significantly more frequently reported in the study population during the COVID-19 pandemic, compared to the pre-epidemic period: “superficial approach to learning”, “distraction”, “mood modification”, “loss of interest”, “isolation”, “sleep disturbances”, “ocular alterations” and “musculoskeletal disorders” (Table 5).

#### Parental attitude towards smartphone use

There were no statistically significant differences, during and before the COVID-19 pandemic, for the following parental attitudes: “surveillance”, “restriction”, “punishment” and “explanation”.By converse, a “permissive” attitude was significantly more frequently observed during the pandemic (Table 5).

### Smartphone addiction

**Fig. 1:**
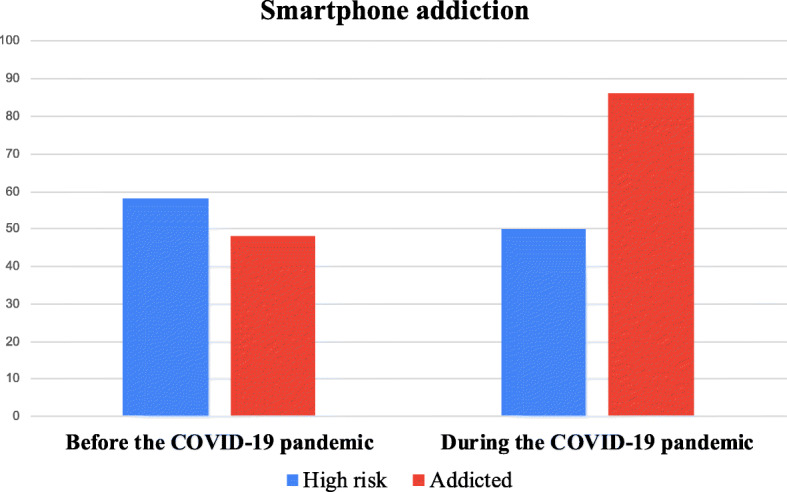
Subjects at high risk and with smartphone addiction, before and during the COVID-19 pandemic

Before the COVID-19 pandemic, among the 184 study participants, 58 (31.5%) were found at high risk of addiction, and in 48 (26.1%) addiction has been documented. During the COVID-19 pandemic, addiction was outlined in 86 (46.7%) participants, while those at high risk were 50 (27.2%) (Fig. 1).

## Discussion

Although the present survey was equally proposed to subjects of both genders, a significantly greater adhesion by females was however observed. This may reflect a higher degree of attention and interest of girls, than boys, to initiatives (including those realized at school) relating to health education. Furthermore, although distributed to subjects belonging to different Italian regions, a wider participation of Sicilian subjects occurred for reasons likely related to direct knowledge of the pediatricians promoting the study. Despite the limitations of an inhomogeneous representation of the population analyzed (either in gender or age or geographic origin), however, the validity of the results obtained may not be affected, also in light of the overlapping conditions of the different Italian regions, in terms of mortality/morbidity rates due to the COVID-19 pandemic, as well as of economic, social and school settings, during the period under investigation. Our study, indeed, showed that, during the pandemic, children and adolescents significantly increased their average daily smartphone use. This may be explained by the wide and multiple role played by smartphones during the epidemic. This is unsurprising, also considering the potential tasks of smartphones as source of communication, information and entertainment [16]. They, indeed, combine the services provided by the web and those of the mobile phone, allowing distance communication and information. At a time of social separation, as that observed in present study, people relied on such useful aspects more than in normal circumstances. Actually, during the COVID-19 pandemic people’s behaviors (and also those of children and adolescents) changed, and partially limited the effects of the crisis [17]. Indeed, smartphones may be used for many purposes, like human connection, learning, and entertainment, and they thus provided psychological and social support. Our study documented an increase, during the pandemic, of smartphone functions like telephone calls, videos, online chats, and social network. This may reflect the lack of physical relationships and contacts with relatives (especially grandparents) and/or friends experienced in this period by children and adolescents, due to the strict social distancing measures adopted [18]. Then, our findings highlighted how during the pandemic communication devices ceased to be only a support for human relationships, and almost entirely replaced face-to-face interactions, becoming the main channel for social connections, especially among young people. Moreover, smartphones provided to children and adolescents the opportunity to continue schooling. Indeed, the partial closure of schools imposed during the pandemic forced teachers to create and use online learning systems [19]. This had pedagogical/psychological consequences in students’ life and families’ organization, due to the need of adequate technological skills, new learning strategies, as well as availability of digital devices and fast connectivity [20, 21]. Online learning led to an increase of the screen time, resulting in turn to a greater possibility of students’ distraction by social media or games [22]. Furthermore, increased digitalization widened the “educational gap” among families. Specifically, poorest children were the most penalized, as they do not likely have a quiet place in their home for their studies, or their own digital devices to access online education [23]. The majority of children and adolescents of present survey, indeed, reported that COVID-19 pandemic affected their learning: online education quality was perceived as lower than in presence one, and pandemic-related anxiety had a negative impact on students’ academic performances [17, 24]. Owing to the temporary and partial closure of educational institutions and suspension of related activities, as well as to the prohibition of shared public spaces, school-age children and adolescents used smartphones more frequently also for entertainment. Indeed, our survey confirmed that their use for recreational purpose became more frequent during the pandemic. The occurrence of infections and/or diseases due to SARS-CoV-2 among family members and/or friends contributed to increase the anxiety levels in children and adolescents, which were related to their variable degree of awareness on risks and consequences of the pandemic [25]. Then, they reported that smartphones allowed to contain negative emotions, stress, sense of isolation and boredom, enabling to avoid, even temporarily, the difficult reality lived during the COVID-19 pandemic and to provide psychological support through different modalities. Specifically, games helped to feel them better and enhance self control, and likewise listening music gave relief from the epidemic-related stressful events [26, 27]. However, by converse, smartphone addiction became more frequent in comparison with the pre-epidemic period. Smartphone overuse may have many negative effects [28, 29]. It may cause cyberbullying, hikikomori, and different neuropsychological and social problems, such as depression, eating and anxiety disorders, low self-confidence, stress, insecurity and solitude, which adversely affect children’s and adolescents’ development and construction of identity [30–34]. School performances may also be negatively influenced by inappropriate use of smartphones, which may lead young people to spend their time unproductively, to be less concentrated and to have a more superficial approach to learning [33, 35]. Smartphone overuse may also lead to adverse clinical outcomes, as observed in our study [36]. Indeed, during the COVID-19 pandemic our children and adolescents had sleep disturbances, ocular alterations and musculoskeletal disorders more frequently than before. Pain on neck, shoulders, wrists and fingers are the musculoskeletal disorders associated to smartphone overuse more frequently reported [37]. They may be influenced by many factors, including smartphone display size, number of messages sent, daily hours spent in front of the screen, and not physiological posture during smartphone use [38]. Their inadequate utilization may also result in ocular problems, such as dry eye disease, irritation and fatigue, burning sensation, conjunctival injection, decreased visual acuity, strain, macular degeneration and acute acquired comitant esotropia [39, 40]. These clinical symptoms may improve limiting smartphone use. This latter is frequent during bed-time among children and adolescents. This is in agreement with our study, in which a major use after midnight at least 3 times per week was observed during the pandemic. The pre-sleep smartphone use may interfere with both sleep duration and quality, and this was reported also in our study [41, 42]. However, sleep disturbances may also be associated with changed sleep-wake habits, which may have independently occurred during the COVID-19 pandemic among children and adolescents.

Finally, parents faced challenging difficulties during the pandemic in the control of their children’s screen time. This was partly likely due to their attempts in finding a complex balance between personal and working life, and family responsibilities [43]. Indeed, in regard to this, a greater parental permissive attitude towards smartphone use was observed, which may have contributed to its major frequency of use during the period under investigation. Parents should set up rules to limit and appropriately use screen time, and oriented to increasingly find moments for effective interactions with their children [44, 45]. This may be crucial for the creation of their emotional and psychological bases, necessary to ensure adequate growth and development.

## Conclusions

Our study showed more frequent smartphone use among Italian children and adolescents during the COVID-19 pandemic, compared to the pre-epidemic period. This may be likely related to the social distancing measures adopted during the months under investigation. The present survey interestingly revealed the changing patterns and aims in the use of smartphones among young people, which allowed to limit some effects of the crisis. Indeed, they were exploited for many purposes such as human connection, learning and entertainment, providing psychological and social support. In the meantime, it was observed a significant increase of overuse and addiction. This led to many unfavorable clinical (sleep, ocular and musculoskeletal disorders), psychological (distraction, mood modification, loss of interest) and social (superficial approach to learning, isolation) outcomes. Pediatricians and health care professionals should be aware of such potential risks related to inappropriate use of smartphones. They should monitor, in cooperation with parents, possible associated adverse effects, in order to early recognize signs and symptoms suggestive, or at high risk, for addiction [46–48]. They must realize, as well, the necessary interventions to prevent and/or lower the detrimental impact of smartphone overuse on children and adolescents’ health, oriented to sustain adequate physical and psychological development as well as social relationships.

## Data Availability

The datasets used and analyzed during the current study are available from the corresponding author on reasonable request.

## Abbreviations

SARS-CoV-2: severe acute respiratory syndrome coronavirus 2
COVID-19: coronavirus disease 2019
DPCM: Decree of the President of the Council of Ministers
SAS-SV: Smartphone Addiction Scale Short Version
SD: standard deviation
WHO: World Health Organization
